# Acquired hemophagocytic syndrome related to parainfluenza virus infection: case report

**DOI:** 10.1186/s13256-015-0552-5

**Published:** 2015-04-08

**Authors:** Nicole Beffermann, Javier Pilcante, Mauricio Sarmiento

**Affiliations:** 1 Universidad Católica de la Santísima Concepción de Chile, Concepción, Chile; 2 Department of Internal Medicine and Hematology and Oncology, Pontificia Universidad Católica de Chile, Lira 85 4to Piso, Santiago, Chile

**Keywords:** Etoposide, Hemophagocytic syndrome, Parainfluenza virus

## Abstract

**Introduction:**

We present the case of a patient with acquired hemophagocytic syndrome secondary to parainfluenza virus infection, a complication that has not, to the best of our knowledge, been previously reported.

**Case presentation:**

A 33-year-old Chilean man with fever secondary to parainfluenza 2 virus infection developed progressive cholestasis, hepatosplenomegaly, cytopenia and an increased ferritin level (>2000IU/L). A bone marrow analysis showed hemophagocytosis. Our patient received HLH-94 chemotherapy, and he achieved complete and sustained remission after a two-year follow-up, without the need for hematopoietic stem cell transplantation.

**Conclusion:**

Hemophagocytic syndrome is a severe disease with high mortality. A high index of suspicion is essential to improve survival. A viral etiology is frequent and although Epstein-Barr virus is the most frequently associated, other viruses like parainfluenza can cause this disease.

## Introduction

Hemophagocytic syndrome (HS) is a highly lethal disease characterized by excessive activation of the immune system with increased levels of cytotoxic lymphocytes and macrophagic activity [1-4]. Characteristic features are the deregulation of critical pathways of the immune response control, an extreme inflammatory state, an increase in cytokine levels, and histiocytic cell hyperactivity [2]. The disease mainly affects children, with an incidence of 1:100,000 live births in the US and 1:50,000 live births in Sweden, with a male to female ratio of 1:1. There are no epidemiological studies in adults; therefore, the disease incidence is unknown [1]. HS is classified as primary if associated with genetic congenital alterations; and secondary if it presents as a consequence of several acquired diseases, such as hematologic malignancies (natural killer leukemia, diffuse large B cell lymphoma, T cell peripheral lymphoma and anaplastic large cell lymphoma), viral infections, rheumatoid arthritis, systemic lupus erythematosus and adult onset Still’s disease. There are cases described in which no specific cause could be identified [1] (Table 1). The most commonly associated viral infection is Epstein-Barr. Other virus that have been related to HS are cytomegalovirus, parvovirus, herpes simplex, varicella zoster, rubella, human herpes virus 8 and human immunodeficiency virus [2-13]. Until now there have been no reports of an association between HS and parainfluenza virus.

**Table 1:** **Acquired causes of hemophagocytic syndrome**

**Infections**	**Autoimmunity**	**Others**
Viral: Epstein-Barr, cytomegalovirus, herpes simplex, varicella, human immunodeficiency virus	Systemic lupus	Common variable immunodeficiency
Bacterial: Brucellosis, Tuberculosis	Still disease	Kawasaki disease
Parasites: Leishmaniasis	Systemic sclerosis	Lymphoproliferative diseases
Fungal	Sjögren’s	Antiretroviral T therapy
	Drug reaction (or rash) with eosinophilia and systemic symptoms syndrome	
	Nodosum polyangiitis	

A diagnosis of HS must be made according to the HLH-2004 trial diagnostic criteria [1,13,14] (Table 2). The most important and frequent features are acute onset of fever, rapidly progressive alteration in liver function tests, hepatosplenomegaly, lymphadenopathy, cytopenia and elevated ferritin levels. A finding of hemophagocytosis is helpful, but not absolutely necessary for diagnosis.

**Table 2:** **Diagnostic criteria for hemophagocytic syndrome according to trial HLH-2004**

**Confirmed molecular disease:**	**PRF1 mutation, UNC13D, Munc 18-2, Rab27a, STX11, SH2D1A, BIRC4**
**Five of eight clinical and laboratory criteria:**	1. Fever
	2. Splenomegaly
	3. Cytopenia affecting two or more cell lines:
	• Hemoglobin <9g/dL
	• Platelets <100,000/mL
	• Neutrophils <1000/mL
	4. Hypertriglyceridemia (fasting triglycerides >265mg/dL or 3mMol/L) and or hypofibrinogenemia (<150mg/dL)
	5. Hemophagocytosis in bone marrow, spleen, liver, lymph node, skin
	6. Decreased or absent natural killer cell activity
	7. Ferritin >500ng/mL^a^
	8. Soluble CD25 >2400U/mL

^a^Ferritin greater than 10,000ng/mL is almost confirmatory.

## Case presentation

A 33-year-old Chilean man presented to our emergency room with a fever of one week’s duration associated with abdominal and back pain. On admission, fever was confirmed (39°C) and findings from a physical examination were normal. Our patient’s daughters had also had a fever with virus-like upper respiratory symptoms one week prior to the onset of our patient’s symptoms.

Initial laboratory tests showed elevated C-reactive protein and a normal cell blood count. Blood and urine cultures showed no infection. There was no alteration in his biochemical serum values. A chest radiography and abdominopelvic computed tomography showed no anomalies. A diagnosis of fever of unknown origin was made and empirical antibiotic therapy (ceftriaxone) was started. Respiratory and enteric viral polymerase chain reaction testing was performed, showing parainfluenza virus-2.

**Figure 1:**
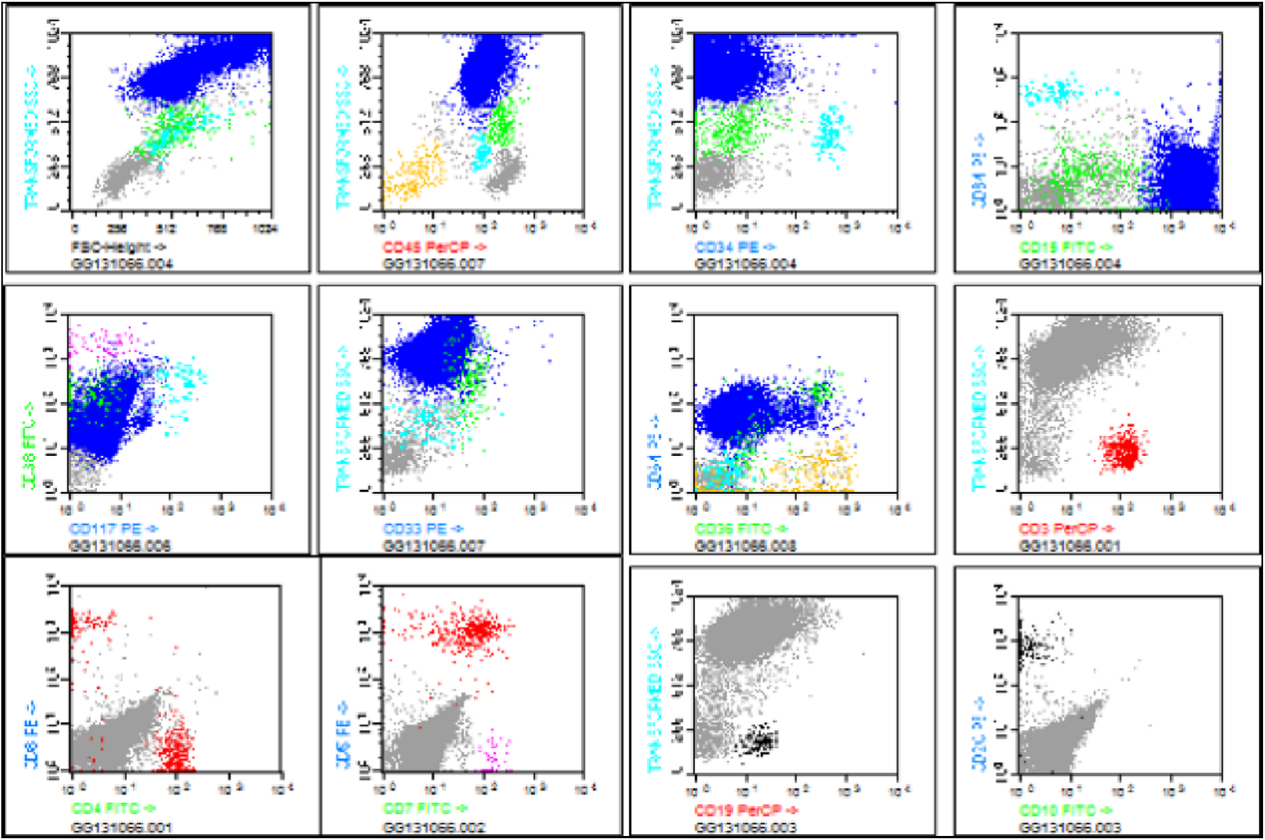
**Bone marrow multiparametric flow cytometry.** Panel multicolor flow cytometry from patient with acquired hemophagocytic syndrome. The first line shows hematopoietic inmunophenotype with normal distribution of CD45, CD34 and CD15; the second line shows an inflammatory pattern of granulocytic population and the third line shows a normal lymphocytic population.

**Figure 2:**
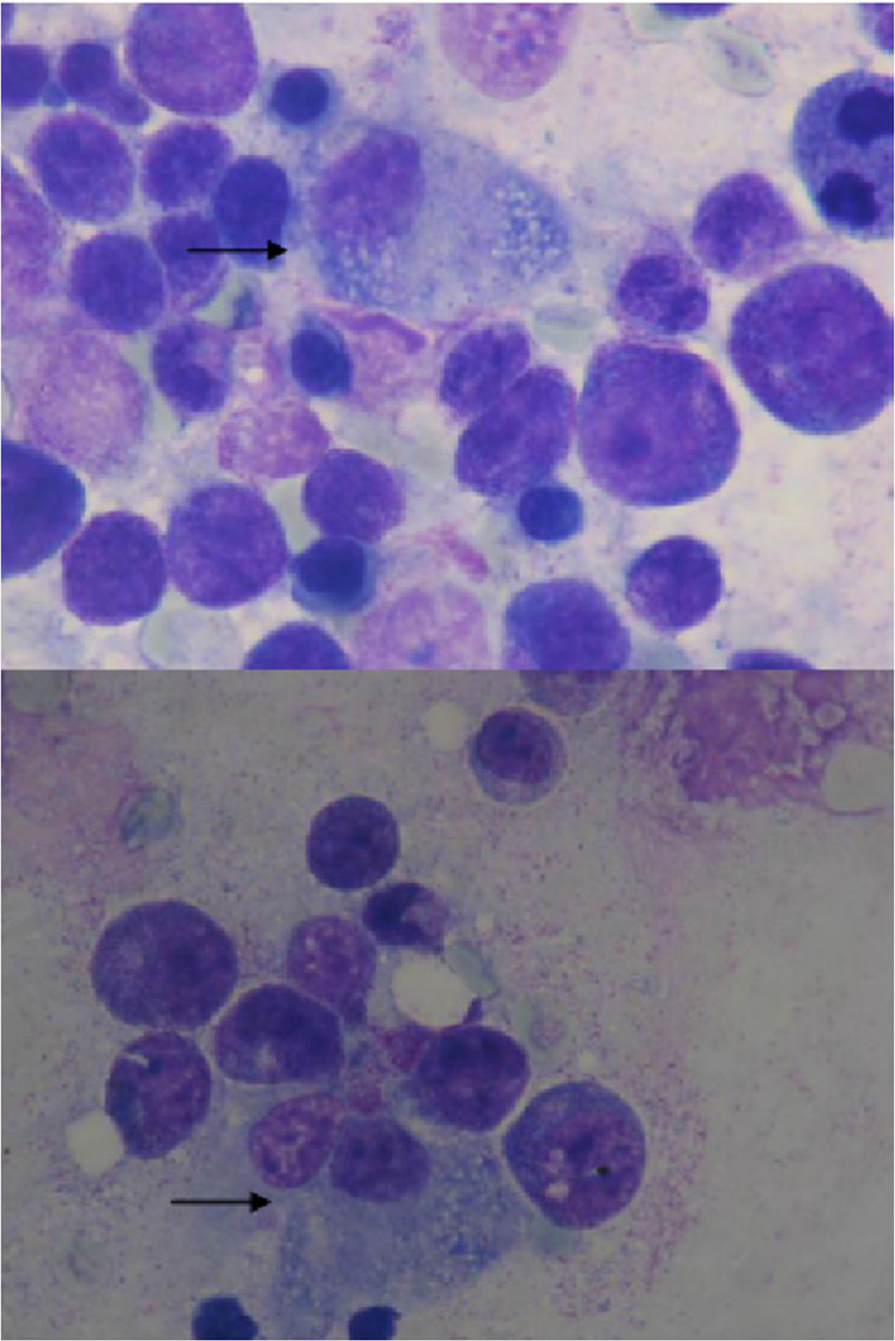
**Bone marrow cytology.** May-Grunwalds/Giemsa-stained bone marrow cytology. The pictures show increased bone marrow cellularity with an hemophagocytosed erythroblast (black arrow).

Five days after admission, our patient developed painful bilateral cervical lymph nodes of 1.5cm, asymmetrical arthralgias in both hands, and an evanescent self-limited rash. Laboratory tests showed he had an elevated erythrocyte sedimentation rate, anemia, and thrombocytopenia (Table 3). Clinical worsening was evident without any response to empirical antibiotic therapy. Noninfectious causes of fever were sought, and we ruled out immunologic diseases and vasculitis. Nine days after admission, we observed an alteration in his liver function test results, which showed a cholestatic pattern (Table 3) with very high ferritin levels (1776IU/L) that increased during the following 24 hours (2352IU/L), and with evident hepatosplenomegaly. Viral hepatitis diagnostic tests were negative. Acquired HS associated with viral infection (parainfluenza 2) was suspected. We performed a bone marrow aspiration study: cytometric analysis showed increased inflammatory cells, concordant with an acute inflammatory process (Figure 1), whereas cytology and examination of a biopsy specimen showed signs of hemophagocytosis (Figure 2). We did not perform a genetic study of primary forms because it was very unlikely that our patient had a genetic disease of delayed presentation.

**Table 3:** **Patient’s laboratory findings**

	**Day of follow-up**					
**Laboratory parameter (normal value)**	**0**	**5**	**9**	**14**	**28**	**365**
White blood cell count (4500 to 11,000/μL)	6500	2200	1800	1100	2000	4900
Hemoglobin (12 to 16gr %)	15	11	10	8	10	16
Platelets (140,000 to 400,000/μL)	245,000	102,000	89,000	9500	130,000	245,000
Alanine transaminase (10 to 40IU)	22	28	93	102	60	18
Aspartate transaminase (10 to 55IU)	35	50	78	75	48	19
Gamma-glutamyltransferase (4 to 50IU)	30	46	115	150	64	22
Ferritin (20 to 390IU/L)			1776	2352	1200	340
Lactate dehydrogenase (135 to 225IU)	100	350	600	490	110	100

Day 0: Hospital admission.

Because of our patient’s rapid clinical progression with diagnostic elements compatible with HS, specific therapy was initiated.

On the 11th day of our patient’s admission, chemotherapy protocol HLH-94 was initiated with intravenous etoposide (150mg/m^2^ twice weekly for two weeks, then once weekly for six weeks), intravenous dexamethasone (10mg/m^2^ daily during weeks one and two, then 5mg/m^2^ daily during weeks three and four, 2.5mg/m^2^ daily during weeks five and six, and finally 1.25mg/m^2^ daily during weeks seven and eight), cyclosporine in doses needed to reach plasmatic levels between 200 and 400μg/dL, and standard prophylactic anti-fungal and antibiotic therapy. A couple of days after the initiation of chemotherapy, our patient showed an acute impairment of consciousness and respiratory failure without coagulopathy, requiring mechanical ventilation. Brain magnetic resonance imaging showed a 45cm^3^ left thalamic hematoma, with insular cortex involvement, mass effect and minimal midline displacement. After his respiratory failure was resolved, global aphasia, right-sided hemiparesis, and swallowing dysfunction were evident. Our patient received physiotherapy and speech therapy, resulting in progressive improvement.

During the second week of chemotherapy, our patient had neutropenic fever, which was treated with broad spectrum antibiotics (cefepime plus vancomycin). Tests revealed a non-fungal lung infection, with a negative etiologic study. Complete resolution was achieved before our patient was discharged.

After initiating the HLH-94 protocol, our patient’s ferritin levels declined gradually. Our patient’s brother was studied for the possibility of an allogeneic hematopoietic stem cell transplantation (HSCT), resulting in human leukocyte antigen full match compatibility. During hospitalization, our patient received two weeks of etoposide and dexamethasone and was discharged to complete outpatient chemotherapy. Cyclosporine was gradually decreased until suspension one year after diagnosis. After two years of follow-up, our patient continues to be in complete remission without the need of a HSCT, with a good quality of life and ferritin levels in normal ranges.

## Discussion

We describe the case of a patient with a common respiratory infection that progressed into acquired HS. Because it was highly unlikely that our patient had an autosomal recessive disorder, which, according to available evidence, would have presented earlier in life (childhood or adolescence), and because laboratory tests ruled out any other cause of macrophage hyperactivation, we believe that the presence of this virus had a pathogenic role in triggering the HS.

Regardless of the cause or trigger, treatment of HS should be based on urgent commencement of chemotherapy and HSCT if necessary. The HLH-94 protocol has achieved an overall survival rate of 55% at 3.1 years in a pediatric population [14,15], and is particularly beneficial in the subgroup of patients without autosomal recessive abnormality of the genes responsible for the disease (heterozygous or homozygous) and with a clear association with viral disease, as in our case. HSCT is mainly performed in patients who are refractory to treatment and in primary forms of the disease, especially in children.

Our patient’s response to HLH-94 was good, with normalization of his ferritin levels and blood cell counts, significant improvement in his neurological deficit, and with no clinical or laboratory evidence of new macrophage hyperactivation. Intrathecal methotrexate was not used because our patient did not present with neurological compromise attributable to macrophage hyperactivation. Our patient’s intracerebral hemorrhage, although concomitant with the clinical course, apparently had no relationship to his HS, especially considering that this syndrome manifests in the central nervous system primarily as encephalitis, seizures or lymphocytic meningitis. However, the association could not be ruled out with certainty.

## Conclusion

HS is a very rare condition that requires a high index of suspicion, because there is a 90% early mortality without specific treatment. Hospitalists should have early suspicion of this lethal disease and achieve an early diagnosis, especially in patients with acquired disease with possible autoimmune or viral causes. Management should be in the hands of a specialist hematological team, with advanced life support in severe cases of HS. In our case, our patient progressed satisfactorily because of a rapid initiation of chemotherapy, optimal management of vital support, and appropriate treatment of his complications. HLH-94 protocol remains the mainstay of treatment [14,15] and has remarkably improved overall survival in acquired HS. In patients with hereditary or congenital forms of the disease, HSCT must be a priority.

## Consent

Written informed consent was obtained from the patient for publication of this case report and accompanying images. A copy of the written consent is available for review by the Editor-in-Chief of this journal.
